# Spectroscopic and AFM characterization of polypeptide-surface interactions: Controls and lipid quantitative analyses

**DOI:** 10.1016/j.dib.2017.03.014

**Published:** 2017-03-12

**Authors:** Øyvind Strømland, Ørjan S. Handegård, Morten L. Govasli, Hanzhen Wen, Samuel Furse, Øyvind Halskau

**Affiliations:** a Department of Molecular Biology, University of Bergen, Bergen, Norway; b Centre for Applied Biotechnology, Uni Research Environment, Bergen, Norway

**Keywords:** AFM, Atomic Force Microscopy, ANTS, 8-Aminonaphthalene-1,3,6-Trisulfonic Acid Disodium Salt, CD, Circular Dichroism, CUBO solvent, Culeddu-Bosco solvent, DLS, Dynamic Light Scattering, DPX, p-Xylene-Bis-Pyridinium Bromide, EYPC, egg yolk phosphatidylcholine, FRET, Förster Resonance Energy Transfer, LUV, Large Unilamellar Vesicles, PBPS, porcine brain phosphatidylserine, NMR, Nuclear Magnetic Resonance, PA, Phosphatidic Acid, PC, Phosphatidylcholine, PC-plas, Phosphatidylcholine plasmalogen, PS, Phosphatidylserine, PS-plas, Phosphatidylserine plasmalogen, SLB, Solid-supported Lipid Bilayers., Lipid bilayers, Chemical degradation, Polypeptide aggregation, Spin-coating, Solid-supported bilayers, Fluorescence, Circular dichroism, Quantitiative ^31^P NMR

## Abstract

This article is related to http://dx.doi.org/10.1016/j.bbamem.2017.01.005 (Ø. Strømland, Ø.S. Handegård, M.L. Govasli, H. Wen, Ø. Halskau, 2017) [1]. In protein and polypeptide-membrane interaction studies, negatively charged lipids are often used as they are a known driver for membrane interaction. When using fluorescence spectroscopy and CD as indicators of polypeptide binding and conformational change, respectively, the effect of zwitterionic lipids only should be documented. The present data documents several aspects of how two engineered polypeptides (A-Cage-C and A-Lnk-C) derived from the membrane associating protein alpha-Lactalbumin affects and are affected by the presence of zwitterionic bilayers in the form of vesicles. We here document the behavior or the Cage and Lnk segments with respect to membrane interaction and their residual fold, using intrinsic tryptophan fluorescence assays. This data description also documents the coverage of solid-supported bilayers prepared by spin-coating mica using binary lipid mixes, a necessary step to ensure that AFM is performed on areas that are covered by lipid bilayers when performing experiments. Uncovered patches are detectable by both force curve measurements and height measurements. We tested naked mica׳s ability to cause aggregation as seen by AFM, and found this to be low compared to preparations containing negatively charged lipids. Work with lipids also carries the risk of chemical degradation taking place during vesicles preparation or other handling of the lipids. We therefor use ^31^P NMR to quantify the head-group content of commonly used commercial extracts before and after a standard protocol for vesicle production is applied.

**Specifications Table**

**Table:** 

Subject area	*Biochemistry, Biophysics*
More specific subject area	*Polypeptide and lipid biochemistry, spectroscopy and biophysical techniques*
Type of data	*Figures*
How data was acquired	*DLS, Far-UV CD,*^*31*^*P NMR, Fluorescence, In-solution tapping mode AFM*
Data format	*Analyzed*
Experimental factors	*Two flexible polypeptides, A-Lnk-C and A-Cage-C, interacting with zwitterionic lipid bilayers at pH 4.5 and pH 7.4. Lipid bilayers prepared by spin-coating onto mica characterized by AFM force measurements in solution. Naked mica subject to polypeptide exposure and imaged using AFM in solution. Quantitative*^*31*^*P NMR acquired on lipid samples suspended in CUBO solvent.*
Experimental features	*CD and fluorescence data on polypeptide-lipid interaction at neutral and acidic conditions. AFM images acquired in tapping mode in buffer. Force measurements performed in contact mode. Lipids vesicles prepared by extrusion and analyzed for chemical degradation using quantitative*^*31*^*P NMR by deconvolution and integration.*
Data source location	*Department of Molecular Biology, Thormøhlensgt. 55, 5008 Bergen; Department of Chemistry, Allegt. 41, 5007 Bergen; Department of Biomedicine, Jonas Lies vei 91, 5009 Bergen. All addresses belong to The University of Bergen, PB 7800, 5020 Bergen.*
Data accessibility	*Data is within this article*

**Value of the data**

• Dynamic light scattering and ^31^P solution NMR can be used to assess the size and lipid composition of LUVs prepared by extrusion.
• Phosphatidic acid is present (at 0.7%) in presumably freshly extracted commercial lipids, and present (at 5.6%) after extrusion to vesicles using standard protocols.
• Atomic force microscopy height- and force-measurements can be applied to characterize lipid bilayers on mica produced by spin-coating.
• Atomic force microscopy data show the interaction of two peptides (pIs 4.2 and 7.0) and bare mica at pH 4.5 and pH 7.4
• Circular dichroism in the far-UV region and a ANTS/DPX FRET-based vesicle leakage assay show the changes in secondary structure and membrane perturbation capability of the two peptides when interaction with zwitterionic vesicles

## 1 Data

**Fig. 1:**
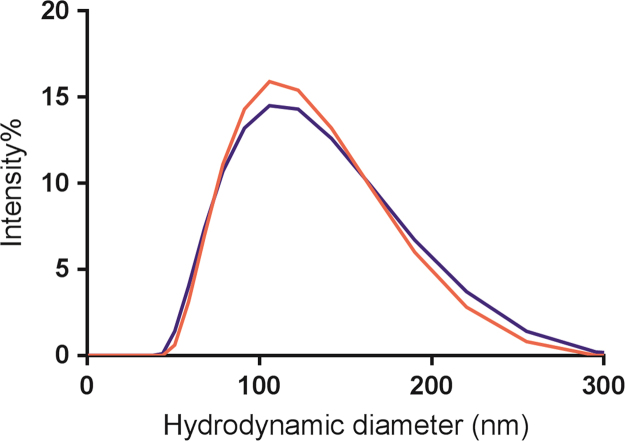
Characterization of EYPC:PBPS LUVs by dynamic light scattering. The graphs show scattered light intensity in relation to hydrodynamic diameter of the vesicles (1 mM total lipid present in cuvette), prepared in buffers at pH 7.4 () and pH 4.5 ().

**Fig. 2:**
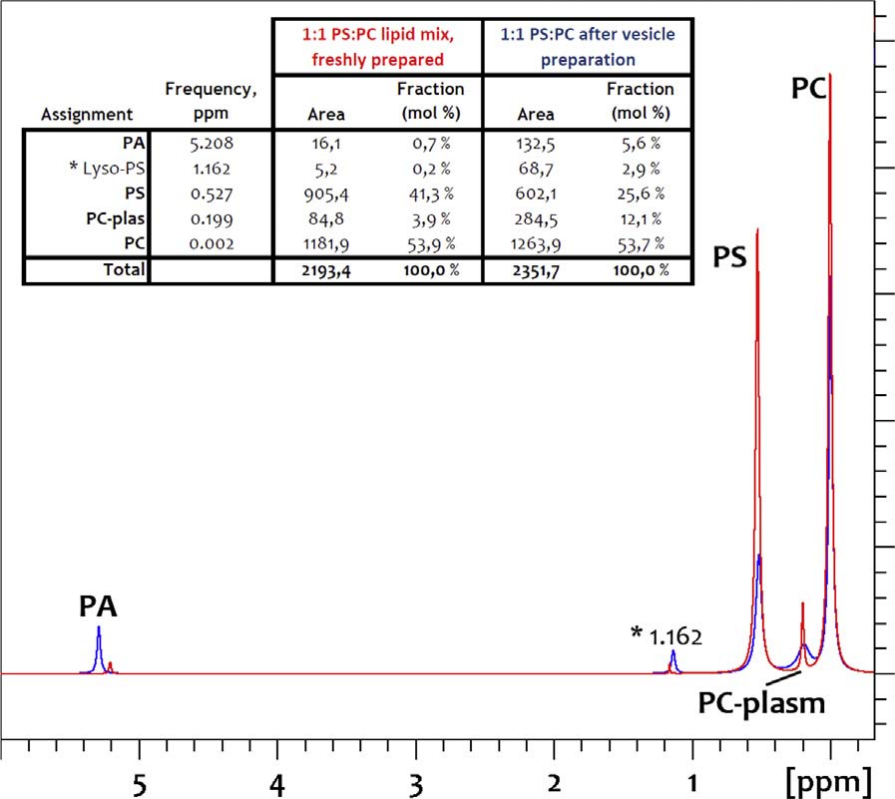
Comparison of lipid headgroup profiles from fresh lipid extracts and from lipid extracts extruded to vesicles. Representative ^31^P NMR traces of phospholipid mixtures (EYPC:PBPS, 1:1 mol %) before () and after () preparation of vesicles (Section 2.1). Lipids are solved in CUBO solvent, at 15 mg/mL lipids, and data acquired as described in Section 2.3. PA: Phosphatidic Acid, PC: phosphatidylcholine, PS, phosphatidylserine, PC-plas: phosphatidylcholine plasmalogen, PS-plas: phosphatidylserine plasmalogen. *1.162 indicates the shift of an unassigned signal, likely lyso-PS. **Insert**: Mol fractions (%) of indicated lipids obtained using the “dcon” function in Topspin 3.5. Chemical shift values correspond to signals for the fresh lipid extracts ().

**Fig. 3:**
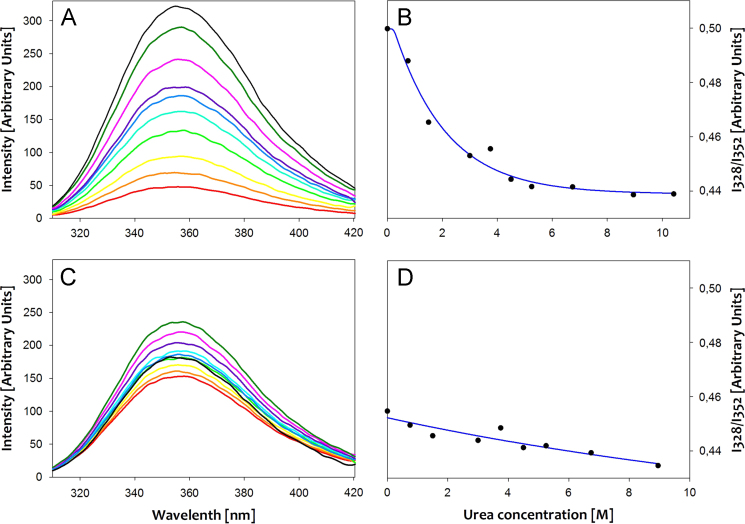
Urea denaturation of Cage-Only (A and B) and Lnk-Only (C and D). The fluorescence spectra are displayed in A and C, where each trace represents increasing amounts of urea present in the cuvette, starting with no urea () through 0.75 M (), 1.5 M (), 3 M (), 3.75 M (), 4.5 M (), 5.25 M (), 6.75 M (), 8.95 M (**––**), and finally 10.45 M (). B and D shows the fluorescence intensity ratio (I328/I352) taken at 328 nm and 352 nm plotted against the urea concentration. The concentration of the peptides was 2 μM for all experiments. The excitation wavelength was set to 290 nm and the experiments were conducted at room temperature in PBS pH 7.4.

**Fig. 4:**
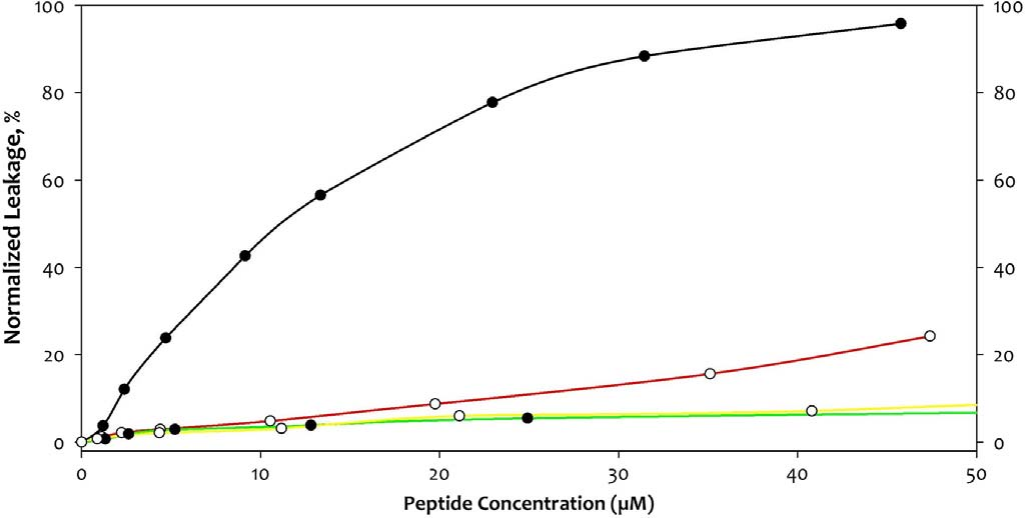
Membrane leakage properties of Cage-Only (**—○—**) and Lnk-Only (**—○—**) vs. A-Cage-C (**—●—**) and A-Lnk-C (**—●—**). Vesicles consisting of EYPC and PBPS lipids (1:1 ratio, 800 μL cuvette volume containing a total of 250 μM lipids) encapsulated with ANTS and DPX were prepared as described above in Section 2.1. The vesicles were subjected to polypeptide concentrations as indicated, and fluorescence emission intensity at 510 nm was recorded. Full ANTS/DPX release from vesicles was achieved by the addition of Triton X-100 (10 µL, 135 mM). Data is plotted as the percentage of the fluorescence intensity at 510 nm before and after addition of Triton X-100 addition. The properties of the A and C helices of the A-Lnk-C and A-Cage-C peptides in relation to lipid interaction are documented in Baumann et al. [13].

**Fig. 5:**
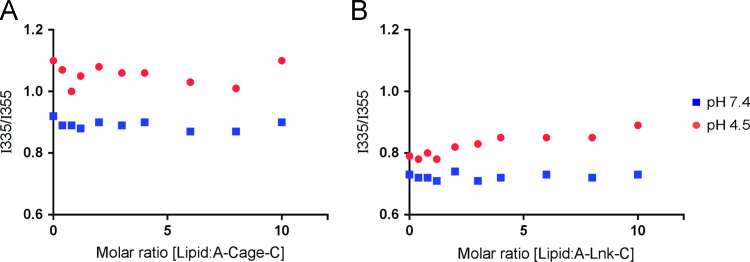
Intrinsic tryptophan fluorescence spectroscopy measurements of A-Cage-C and A-Lnk-C upon association with zwitterionic membranes. The interaction of A-Cage-C (A) and A-Lnk-C (B) at the indicated pH values with increasing amounts of EYPC LUVs is shown as the ratio of fluorescence intensities at 335 nm/355 nm (excitation at 295 nm). The cuvette peptide concentration was 5 μM.

**Fig. 6:**
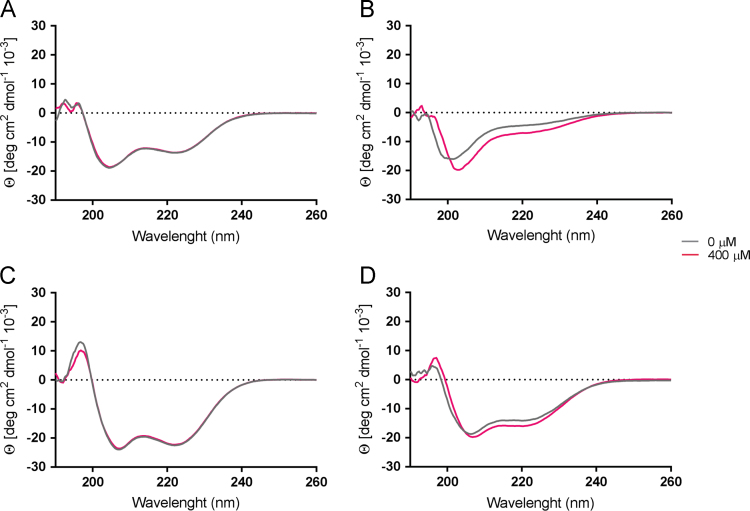
pH-dependent changes to the secondary structure of A-Cage-C and A-Lnk-C upon association with zwitterionic membranes monitored by CD. CD spectra of A-Cage-C at pH 7.4 (A) and pH 4.5 (C), and A-Lnk-C at pH 7.4 (B) and pH 4.5 (D) in the absence () and presence () of 400 µM EYPC LUVs, in terms of the total lipid concentration. The cuvette peptide concentration was 50 μM.

**Fig. 7:**
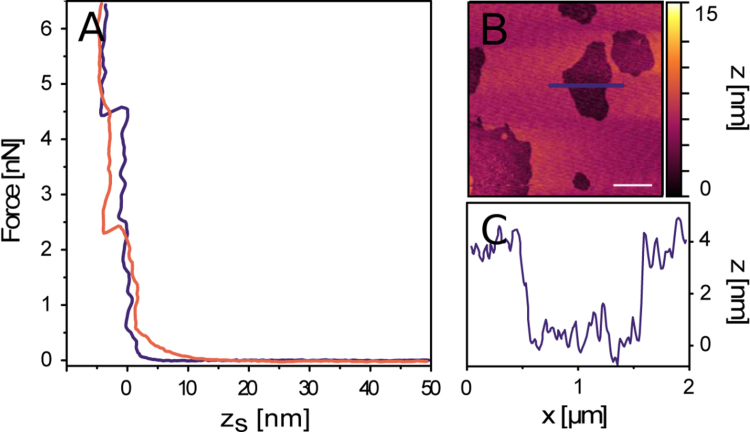
Verification of supported lipid bilayers using AFM cantileverpenetration force measurements and height differences. A) The change in force exerted by the cantilever as the tip approaches and bends at the surface of the substrate. In both curves, representing neutral and acidic pH, a “jump” occurs of lengths close to that of a typical lipid bilayer. B and C) Height traces from an AFM image showing the height difference between mica and lipid-covered surfaces.

**Fig. 8:**
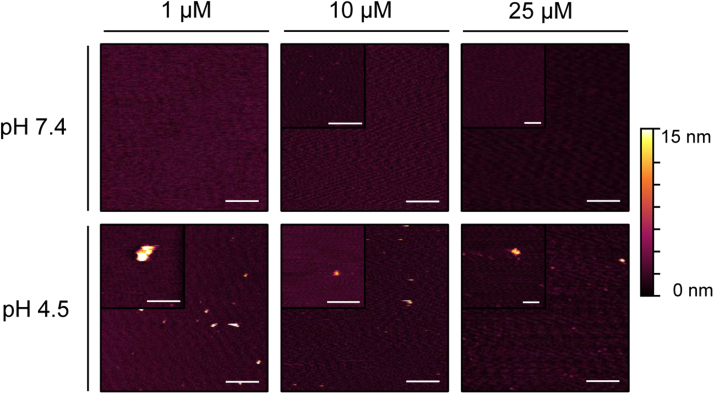
Association of A-Cage-C on naked mica imaged by AFM in solution. AFM z-sensor images were acquired of A-Cage-C, with the indicated concentrations, at pH 7.4 (upper panels) on mica. Subsequently, the pH was lowered to pH 4.5 and images were acquired (lower panels) All images were acquired in tapping mode, and subjected to standard flattening procedures using Gwyddion. Scale bars are 1 µM and 200 nm for the insets, respectively. The height color gradient is 0–15 nm for all images.

**Fig. 9:**
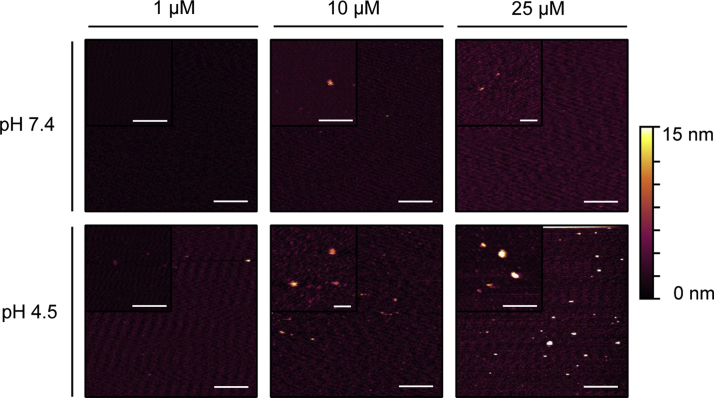
Association of A-Lnk-C on naked mica imaged by AFM in solution. AFM z-sensor images were acquired of A-Lnk-C, with the indicated concentrations, at pH 7.4 (upper panels) on mica. Subsequently, the pH was lowered to pH 4.5 and images were acquired (lower panels) All images were acquired in tapping mode, and subjected to standard flattening procedures using Gwyddion. Scale bars are 1 µM and 200 nm for the insets, respectively. The height color gradient is 0–15 nm for all images.

The size distributions of large unilamellar vesicles (LUVs) prepared by extrusion are presented in (Fig. 1). Solution phase ^31^P NMR was used to determine the lipid compositions of the LUVs and to elucidate whether modulations in lipid composition occurred during vesicle preparation (Fig. 2). The fold and membrane perturbation capability of the linking segments, Cage-only and Lnk-Only, from the A-Lnk-C and A-Cage-C peptides was investigated by urea denaturation and a Förster Resonance Energy Transfer (FRET) -based membrane leakage assay (Fig. 3, Fig. 4). The tryptophan environment and secondary structure of A-Lnk-C and A-Cage-C in the presence and absence of zwitterionic LUVs is shown in (Fig. 5, Fig. 6). Then, the efficacy of depositing lipid bilayers on mica by spin-coating was elucidated by Atomic Force Microscopy (AFM). The presence or absence of bilayers were assessed by probing the prepared surfaces using the tip of the AFM cantilever (Fig. 7A), and height-profiling of the bilayers (Fig. 7BC). Finally, the aggregation of the polypeptides at neutral and acidic conditions in the presence of mica was investigated by AFM in solution (Fig. 8, Fig. 9).

## 2 Experimental design, materials and methods

### 2.1 Materials

A-Lnk-C (EQLTKAEVFRELKDLKGYVGRAWKAGVISAAKFLDDDLTDDIMAVKKILDKVG) and A-Cage-C (EQLTKAEVFRELKDLNLYIQWLKDGGPSSGRPPPSFLDDDLTDDIMAVKKILDKVG) were obtained using recombinant expression in *E. coli* as described in [1]. Cage, based on a Trp-Cage fold (NLYIQWLKDGGPSSGRPPPS, molecular weight 2169 Da, theoretical pI=8.59, [3], and Lnk, based on a random basic sequence (KGYVGRAWKAGVISAAK, molecular weight = 1762 Da, theoretical pI=10.46), were produced by CPC Scientific using solid-phase Fmoc/t-Boc synthesis. Egg yolk phosphatidylcholine (EYPC) and porcine brain phosphatidylserine (PBPS) were obtained from Avanti Polar Lipids. AFM cantilevers TR400PSA were obtained from Olympus and Mica grade V1 were obtained from Electron Microscopy Sciences. All other reagents were purchased from Sigma-Aldrich.

### 2.2 Vesicle preparation

Briefly, vesicles consisting of EYPC and PBPS lipids (1:1 ratio, for DLS measurements, leakage assays and quantitative ^31^P NMR) or EYPC only (CD and intrinsic fluorescence assays) were prepared by extrusion of hydrated multilamellar lipid aggregates using a protocol adapted from [2]. The hydrated multilamellar lipids were prepared as follows: In a glass vial covered in foil, appropriate amounts of lipids, dissolved in chloroform, was added. The solvent was evaporated using a stream of N_2_ gas, producing a lipid film. After addition of buffer at pH 4.5 (5.4 mM citric acid, 6.4 mM Na_2_HPO_4_, 50 mM NaCl) or 7.4 (16 mM K_2_HPO_4_, 4 mM KH_2_PO_4_, 50 mM NaCl), the solution was flushed with N_2_ gas, stoppered, and incubated overnight at 37 °C and 250 rpm. Finally, the hydrated lipid mixture was subjected to freeze-thawing in liquid N_2_ (−196 °C) and water (60 °C) for 6 cycles. A Mini Extruder (Avanti Polar Lipids) was used to pass the hydrate lipid mixture through a double polycarbonate filter (100 nm pore size, Whatman) 9 times, and its visual appearance changed gradually from milky to transparent during this process. For a description of how to assemble, use, and clean the extruder, please refer to [3]. The extruded solutions was collected in a foil-wrapped glass tube, flushed with N_2_ gas, and used the same or following day for experiments. Total lipid concentration was 1 mM for both neutral and acidic pH vesicle suspensions. To perform FRET-based leakage assays, it is necessary to include a fluorophore (8-aminonaphtalene-1,3,6,-trisulfonic acid, ANTS) and a quencher (p-xylene-bis-N-pyridinium bromide, DPX) into the vesicles. For the preparation of LUVs with ANTS and DPX encapsulated, vesicles were produced as above, except that the buffers in question also contained 12.5 mM ANTS and 45 mM DPX. After the final extrusion step, the unencapsulated ANTS and DPX was removed by gel-filtration using a PD-10 column (gravity protocol, GE Healthcare).

### 2.3 Dynamic light scattering

The size distribution of the LUVs was determined by Dynamic Light Scattering using a Zetasizer Nano ZS (Malvern Instruments). The following settings were used: Material Refractive Index: 1.45; Solvent Refractive Index 1.333; Viscosity, 0.9238; Scatter Angle 173°. Experiments were performed at 25 °C using an equilibration time of 10 s, and 20 runs with an individual run duration of 20 s. The lower and upper threshold setting was 0.1 nm and 6000 nm, respectively.

### 2.4 Quantitative ^31^P NMR of the lipid extracts

Samples of the lipid mixtures were collected from both lipid films and from vesicles. The latter were freeze-dried before preparation of the NMR samples. The dried lipid mixtures were dissolved using the CUBO (Culeddo-Bosco) solvent system, consisting of 1 ml dimethylformamide and 0.3 mL trimethylamine by volume, and 100 mg guanidinium chloride [4], [5]. 500 µL of CUBO solvent volume and ca. 15 mg/mL lipid per sample were used, as this setup dissolve all lipid species and provide good signal dispersion for quantitative ^31^P NMR [6]. 1D ^31^P NMR was performed on a Bruker AV500 MHz instrument fitted with a BBO probe at room temperature. Acquisition parameters were as follows: TD: 32k, ns: 4196, ds: 2, d1: 4s, SW: 19.98 ppm, and proton decoupling was achieved using the Waltz-16 pusleprogram. The spectra were processed in Topspin applying 1.5 Hz line broadening to the FID prior to Fourier transformation using for deconvolution (the “decon” function in Topspin) and peak integration for signal quantification. Each signal was assigned a percentage of the sum of all phospholipid integrations.

### 2.5 ANTS-DPX fluorescence monitored leakage assays

FRET-based vesicle membrane integrity assays were performed to determine whether Cage-only or Lnk-only were able to induce leakage comparable to A-Cage-C and A-Lnk-C in LUVs. Vesicles consisting of EYPC and PBPS lipids (1:1 ratio) with ANTS and DPX encapsulated were prepared as described above in Section 2.1. At high concentrations inside the vesicles, the ANTS/DPX fluorophore/quencher pair will have a low fluorescence response. If the vesicle bilayer is perturbed by membrane active substance, ANTS and DPX will be released and diluted. DPX can then no longer effectively quench ANTS, and there will be an increase in the fluorescence of this substance [7]. Initial samples of 800 μL containing 250 μM EYPC:PBPS were prepared and A-Lnk-C, A-Cage-C, Cage-only or Lnk-only were each added stepwise between the measurements up to 40 µM of peptide concentration. Then, the detergent Triton-X (10 μL of 135 mM stock solution) was added to the cuvette to release all remaining ANTS and DPX.

Fluorescence spectroscopy was carried out using a LS50B florescence spectrometer (Perkin Elmer). Samples were excited at 355 nm and scanned from 450 nm to 550 nm, using slit widths of 5 nm, a scan speed of 200 nm min^−1^, and 3 averaged scans per experiment. The experiments were carried out at 25 °C. Buffer only was used as blanks and subtracted using the FL WinLab software. In the data analysis the measured intensities were adjusted to account for the dilution as protein were added using a simple linear relationship. The measurements containing only LUVs in buffer were arbitrarily set as 0% leakage and the measurements where Triton-X was added as 100%, based on the intensities at 510 nm which is the at λ_max_ of ANTS.

### 2.6 Urea denaturation and intrinsic tryptophan fluorescence spectroscopy

Each peptide investigated contains one single tryptophan in their sequence. Tryptophans are fluorophores sensitive to changes in the polarity of the local environment and thus a useful reporter on whether it is protected from the solvent by a fold or embedment into a lipid bilayer. Intrinsic tryptophan fluorescence spectroscopy was performed using a LS50B florescence spectrometer (Perkin Elmer). The excitation wavelength was set to 295 nm (290 nm in the case of urea denaturation experiments, vide infra) and emission was measured from 310 nm to 380 nm using a scan rate of 25 nm/min, and the slit width was 5 nm for both excitation and emission. Samples of 5 µM peptides at either pH 4.5 (5.4 mM citric acid, 6.4 mM Na_2_HPO_4_, 50 mM NaCl) or 7.4 (16 mM K_2_HPO_4_, 4 mM KH_2_PO_4_, 50 mM NaCl) were prepared in the presence and absence of various concentrations of EYPC LUVs and loaded in a cuvette (Hellma 104-QS, light path-length 10 mm) and scanned three times. In the case of urea denaturation, experiments were performed in buffer at pH 7.4 (16 mM K_2_HPO_4_, 4 mM KH_2_PO_4_, 50 mM NaCl) and increasing amounts of urea (0–10.45 M). Polypeptide concentration was 2 µM. All samples were blank corrected using appropriate blank samples. Data was plotted using the approach described in Mårtensson et al. [8]. Briefly, a wavelength corresponding to a tryptophan in a hydrophobic environment and an exposed tryptophan environment is picked. The intensity of the hydrophobic wavelength (usually picked between 320 nm–335 nm) is divided by the intensity at the wavelength of the exposed situation (usually picked between 350 nm–355 nm). Since fluorescence emission produced very broad spectra, this ratio is used as a useful proxy variable for tracking changes in the spectrum, instead of relying on visual inspection of very broad and overlapping peaks.

### 2.7 Circular dichroism spectroscopy

In order to track changes in secondary structure, CD spectroscopy in the far-UV region (190–260 nm) was performed. For reviews on the application of CD to polypeptides, please see [9], [10]. The experiments were carried out on a Jasco J-810 spectropolarimeter (Jasco Analytical Instruments fitted with a Peltier element for temperature control), and experiments were performed at 25 °C. Samples with 50 µM peptide and various concentrations of EYPC LUVs at pH 4.5 (5.4 mM citric acid, 4.6 mM Na_2_HPO_4_, 50 mM NaCl) or 7.4 (16 mM K_2_HPO_4_, 4 mM KH_2_PO_4_, 50 mM NaCl) were loaded in a 1 mm quartz cuvette (Hellma 110-QS). Each sample was scanned three times at 50 nm/min using a bandwidth of 0.2 nm, and a data pitch of 0.1 nm. The recorded HT voltage and absorption showed that the samples had low levels of light scattering and absorption down to 200 nm and tolerable down to 190 nm. The data series were acquired using the same peptide stocks. Their concentration was determined by UV–vis absorption at 280 nm [11], using a NanoDrop ND-1000 Spectrophotometer. The data was blank corrected using the instrument manufacturers’ software, and converted from optical rotation to mean residual ellipticity (ϴ) using the equation ϴ=*ɛ*/10·*C*·*n*·*l*). Here, *ɛ* is the measured ellipticity, *l* is the path length (cm), *C* is the determined peptide concentration (M), and *n* is the number of amino acid in the peptides, which are 53 (A-Lnk-C), 56 (A-Cage-C).

### 2.8 Atomic force microscopy

AFM data was acquired using a MFP-3D-Bio (Asylum Research) instrument equipped with a TR400PSA1 (Olympus) cantilever. Imaging was performed in tapping mode and force curves were obtained in contact mode, operating in liquid environment using a Fluid Cell Lite (Asylum Research) at room temperature. The samples were hydrated for at least 1 h in buffer (150 mM NaCl, 10.1 mM Na_2_HPO_4_ and 0.9 mM citric acid, pH 7.4) before imaging and injection of peptide, having a total volume of 1.5 mL. Lowering pH of buffer from neutral to acidic was achieved by titration using a solution of 150 mM NaCl, 100 mM citric acid. The AFM data was processed using Igor Pro (Wavemetrics) and Gwyddion [12].

### 2.9 Force measurements

Forces between the AFM tip and the sample surface can be approximated using Hooke׳s law, and depend on the surface probed, the cantilever tip material and the medium in which the experiment takes place. Force measurements can therefore be used to differentiate between different surface types within the same setup. To estimate the force between the cantilever tip and a given surface, the spring displacement, *Δx*, is substituted by a term for cantilever displacement. This displacement is calculated as the slope of cantilever deflection when its tip is pushed against an atomically flat surface. Using the slope of contact region, usually referred to as the detection inverse optical lever sensitivity (invOLS), the force can be calculated using a modified Hooke׳s law:

$$F=k\Delta x=k\left(s_{d}V_{d}\right)=k([\Delta V_{d}/\Delta z]\cdot V_{d})$$

where *k* is the cantilever spring constant, *z* is the piezoelectric z-sensor position, *s*_*d*_ is the slope of contact region (deflection invOLS), *V*_*d*_ is the detection voltage. AFM force measurements are usually plotted as z-sensor position versus force.

The samples subjected to AFM force measurements were prepared using a EYPC:PBPS lipid mixture which was spin-coated on grade V1 mica (Electron Microscopy Sciences). To control for the presence of lipid bilayers at a given point of mica-supported bilayers, the cantilever tip was pressed onto the surface in contact mode. If lipids are present, the cantilever will push through the bilayer when a certain critical force is attained. This breakthrough can be observed as abrupt leap in force-distance plots.
